# Associations of dietary choline and betaine with all-cause mortality: a prospective study in a large Swedish cohort

**DOI:** 10.1007/s00394-023-03300-y

**Published:** 2024-01-04

**Authors:** Therese Karlsson, Anna Winkvist, Anna Strid, Bernt Lindahl, Ingegerd Johansson

**Affiliations:** 1 Department of Internal Medicine and Clinical Nutrition, Institute of Medicine, Sahlgrenska Academy, University of Gothenburg, P. O. Box 459, S-405 30 Gothenburg, Sweden; 2 Department of Life Sciences, Division of Food and Nutrition Science, Chalmers University of Technology, Gothenburg, Sweden; 3 Department of Public Health and Clinical Medicine, Sustainable Health, Umeå University, Umeå, Sweden; 4 Department of Odontology, Umeå University, Umeå, Sweden

**Keywords:** Choline, Phosphatidylcholine, Betaine, Mortality, Prospective cohort, Västerbotten Intervention Programme

## Abstract

**Purpose:**

Investigate the association between choline and betaine intake and all-cause mortality in a large Swedish cohort.

**Methods:**

Women (52,246) and men (50,485) attending the Västerbotten Intervention Programme 1990–2016 were included. Cox proportional hazard regression models adjusted for energy intake, age, BMI, smoking, education, and physical activity were used to estimate mortality risk according to betaine, total choline, phosphatidylcholine, glycerophosphocholine, phosphocholine, sphingomyelin, and free choline intakes [continuous (per 50 mg increase) and in quintiles].

**Results:**

During a median follow-up of 16 years, 3088 and 4214 deaths were registered in women and men, respectively. Total choline intake was not associated with all-cause mortality in women (HR 1.01; 95% CI 0.97, 1.06; *P* = 0.61) or men (HR 1.01; 95% CI 0.98, 1.04; *P* = 0.54). Betaine intake was associated with decreased risk of all-cause mortality in women (HR 0.95; 95% CI 0.91, 0.98; *P* < 0.01) but not in men. Intake of free choline was negatively associated with risk of all-cause mortality in women (HR 0.98; 95% CI 0.96, 1.00; *P* = 0.01). No other associations were found between intake of the different choline compounds and all-cause mortality. In women aged ≥ 55 years, phosphatidylcholine intake was positively associated with all-cause mortality. In men with higher folate intake, total choline intake was positively associated with all-cause mortality.

**Conclusion:**

Overall, our results do not support that intake of total choline is associated with all-cause mortality. However, some associations were modified by age and with higher folate intake dependent on sex. Higher intake of betaine was associated with lower risk of all-cause mortality in women.

**Supplementary Information:**

The online version contains supplementary material available at 10.1007/s00394-023-03300-y.

## Introduction

Choline and betaine are two metabolically related nutrients involved in several important physiological functions in the human body. Choline is either consumed through diet or produced via endogenous de novo synthesis [1, 2]; however, endogenous synthesis is insufficient to prevent deficiency and choline is therefore considered an essential nutrient [3]. The main dietary sources of choline include animal-based foods, such as meat, dairy, and egg [4]. Betaine, in addition to originating from consumption of plant-based foods, such as cereals and cereal products [5], can be synthesized from choline in an irreversible reaction [1].

Higher intake of choline and betaine has been associated with decreased circulating concentrations of homocysteine (Hcy) [6, 7]. Elevated systemic concentrations of total Hcy have been linked to cardiovascular disease (CVD) [1]. Furthermore, higher intake of choline and betaine has been associated with decreased circulating concentrations of inflammatory markers [8]. Therefore, higher choline and betaine consumption may be beneficial to health. Conversely, dietary choline and betaine have been hypothesized to have harmful effects via their intestinal production of trimethylamine-N-oxide (TMAO); TMAO has been shown to increase the risk of all-cause mortality in prospective cohort studies [9, 10]. Several B-vitamins act as co-factors in some of the aforementioned reactions, and the presence of folate or vitamin B-12 deficiency has been shown to increase choline and betaine requirements [1]. Therefore, interrelationships of B-vitamins and choline and betaine intake should be considered when assessing these diet–health relationships.

Epidemiologic evidence on associations between intake of choline and betaine and mortality is inconsistent. Choline intake has been positively associated with all-cause mortality in two large U.S cohorts [11, 12]. In three ethnically diverse U.S and Chinese cohorts, higher intake of total choline was associated with increased risk of CVD mortality in black Americans and Chinese populations but not in white Americans [13]. A prospective study of Japanese adults reported null associations between choline and betaine intake and CVD mortality [14]. In a 2019 meta-analysis, choline and betaine intake was not associated with cancer survival [15]. Prospective studies on relationships between choline and betaine intake and all-cause mortality in European populations are lacking. The risk for chronic diseases as well as nutritional needs based on physiological and hormonal factors differ between women and men [16]. Particularly, choline requirements are higher in men and post-menopausal women compared to in pre-menopausal women [17].

The aim of the present study was to investigate associations between intake of choline and betaine and total mortality in a large population-based cohort in northern Sweden. The large cohort enabled us to analyze risk for total mortality in women and men separately. Also, interactions between choline and betaine intake and intake of B-vitamins on mortality risk were explored.

## Methods

### Study population and design

The Västerbotten Intervention Programme (VIP) is an ongoing population-based prospective cohort study (start in 1985). Residents in the Västerbotten County in northern Sweden are invited to a health screening when they turn 40, 50, and 60 years, and until 1996 also 30 years of age. Participants go through a comprehensive health screening, fill out lifestyle questionnaires, and donate blood samples. The participation rate has varied between 48 and 67% over the years [18], with little evidence of selection bias [19].

**Fig. 1:**
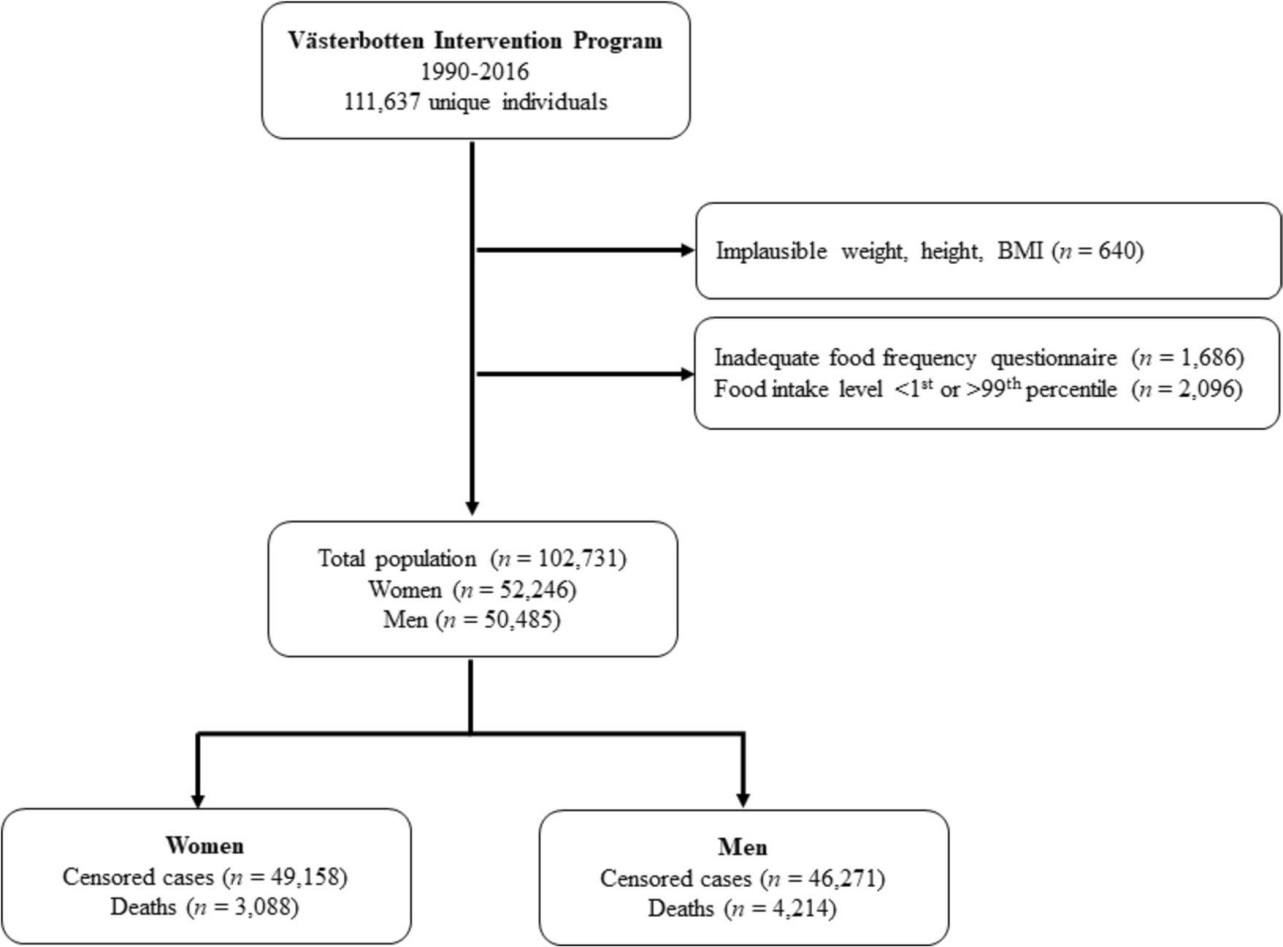
Flowchart of study participants from inclusion of the Västerbotten Intervention Programme to the final study groups

Some participants have had more than one visit in VIP. For the current study, data from the first visit only were included. Between 1990 (when questionnaires became optically readable) and 2016, in total, 111,637 unique individuals had at least one recorded visit in VIP. Among these, individuals with implausible weight < 35 kg, length < 130 cm or body mass index (BMI) < 15 were excluded (*n* = 640). Furthermore, exclusions due to incomplete food intake reports and extreme energy intakes were made: individuals with > 10% of the food-frequency questionnaire (FFQ) items missing (*n* = 1686), individuals who lacked portion size indications (*n* = 4484), and individuals with food intake level (FIL; calculated by dividing reported total energy intake with estimated basal metabolic rate [20]) below the 1st percentile or above the 99th percentile calculated separately by sex or missing body weight, so that FIL could not be calculated (*n* = 2096). In total, 102,731 individuals were included in the current analysis (Fig. 1).

### Dietary assessment

The Northern Sweden Diet Database (NSDD) consists of dietary data collected in VIP. Habitual dietary intake, reflecting intake during the previous year, was initially assessed by an 84-item semi-quantitative FFQ. From 1996, a 64–66-food item FFQ was used. The reduction was achieved by deleting food less commonly consumed or in a few cases merging of foods with similar nutrient profiles. The FFQ was completed at the health screening. Alternatives of frequency ranged from never to four times or more per day. To estimate portion sizes, the FFQ includes four pictures of increasing portion sizes for staple foods (rice/potatoes/pasta), meat/fish, and vegetables. For other foods, age- or sex-specific portion sizes or natural sizes (e.g., a fruit) were used [18]. Daily nutrient intakes (not including dietary supplements) were calculated using the national food composition database [21]. The longer version of the FFQ has been compared against repeated 24-h recalls and circulating biomarkers [22, 23]. These comparisons showed that the FFQ had good reproducibility and that correlations of nutrients and foods with the 24-h recalls were of similar size as in other cohort studies [22].

Intake of total choline as well as individual forms of choline and betaine was calculated using the U.S. Department of Agriculture (USDA) Database for the Choline Content of Common Foods, release 2 [24]. Only a few later updates (2015) of choline content to the original database were included (https://fdc.nal.usda.gov/) and one additional reference source for choline content in cream was used [25]. Total choline was calculated as the sum of free choline, phosphatidylcholine, glycerophosphocholine, phosphocholine, and sphingomyelin. If a food item did not occur in the USDA database, a nutritionally equivalent food was used for estimating choline and betaine content (ex. English muffin for sweet wheat bread/wheat cracker for flat bread). For dishes not existing in the USDA database, choline and betaine content was calculated for each ingredient in the recipe used in the FFQ. Early versions of the FFQ did not include a specific question on whole egg intake. Based on reported egg intake from FFQs that included a question on whole egg intake, median intake was imputed for missing data (32.6%).

### Covariates and baseline data

At the study visit, participants donated a fasting (≥ 4 h) venous blood sample and filled in an extensive lifestyle questionnaire. Blood pressure was measured after a 5 min rest. A benchtop analyzer was used to analyze blood glucose concentrations. Initially, total serum cholesterol was analyzed at health centers using a Reflotron benchtop analyzer (Boehringer Mannheim GmbH, Diagnostica), whereas after 1 September 2009, an enzymatic routine method was used at the Clinical Chemistry Department at the nearest local hospital. An algorithm from a calibration set was used to perform harmonization [18]. Smoking status was based on self-reported data and for the present analyses categorized into current smoker, ex-smoker and non-smoker. Physical activity was estimated using the Cambridge index of Physical activity [26] and thus categorized into inactive, moderately inactive, moderately active, and active. Educational level was collapsed into three categories (basic level of 9 years, senior high school, and university). Body weight (kg) and height (m) were measured in light clothing without shoes using standardized weight and measuring scales. BMI was calculated as kg/m^2^.

### Follow-up and clinical endpoints

Fatal events were identified by linking the Swedish Cause of Death registries at the National Board of Health and Welfare/Socialstyrelsen (https://www.socialstyrelsen.se/statistik-och-data/register/) to the personal identification number of the VIP participants. The primary endpoint was all-cause mortality. Study participants were followed from enrollment until death or 31 December 2016, whichever occurred first.

### Statistics

All analyses were performed separately for women and men. Continuous variables are presented as mean (SD) and categorical variables as counts (percentages). Choline and betaine intake were energy-adjusted using the residual method, while other dietary intake variables were energy-adjusted using the residual (micronutrients) or density method (macronutrients) [27]. Dummy variables were created for missing values for education, smoking, and physical activity. Spearman’s rank correlation was used to evaluate correlations between total choline and the different forms of choline. Comparison of baseline characteristics and diet by quintiles of choline intake were performed using linear regression (adjusted for age and year of study participant) for continuous data and Pearson Chi-square test for categorical data.

Hazard ratios (HR) with 95% confidence intervals (CI) for the association between intake of choline or betaine and all-cause mortality were calculated by Cox proportional hazard regression. The primary analyses were performed using quintiles with the lowest quintile as reference group. A p value for trend was calculated by including the quintile (1–5) of choline or betaine intake as a numeric variable. Analyses of choline or betaine intake as continuous variables were also performed and reported as hazard ratios per daily increment of 50 mg/day of total choline, phosphatidylcholine, and betaine, and 5 mg/day for the remaining choline forms. Non-linear relationships of total choline and betaine intake with all-cause-mortality were explored using restricted cubic splines with five knots at the 5th, 27.5th, 50th, 72.5th and 95th percentiles [28]. Time in months between baseline and death or end of the study period was used as the time scale. The proportional hazards assumption was tested using Schoenfeld residuals. No violation of proportional hazard assumptions was observed in men. In women, age expressed a time trend and was treated as time-dependent covariate in all models. Potential confounding variables were identified a priori, based on similar analyses in the previous literature. The basic models included reported energy intake and age and adjusted models also included BMI, smoking status, educational level, and physical activity.

Effect modifications were studied according to age groups and subgroups based on high or low (dichotomized according to median value) folate, vitamin B-6, vitamin B-12, animal protein, and whole grain intakes. Statistical testing of effect modification was performed by adding interaction product terms of choline or betaine and B-vitamin intakes in the final Cox-regression model; these were evaluated at alpha level 0.10.

The computer software packages SPSS for Windows, version 28 (IBM, NY, USA), Stata Statistical Software: Release16 (TX: StataCorp LLC), and R version 4.3.2 (The R foundation for Statistical Computing, Vienna, Austria) were used for statistical analyses. Non-linear Cox-regression analyses were implemented using the rms version 6.7–1 package in R. A two-sided *P* value < 0.05 was considered statistically significant.

## Results

### Baseline characteristics and diet

Overall, there were 52,246 women and 50,485 men from the VIP included in the current analysis. Mean (SD) reported intakes of total choline were 255 (48.6) and 278 (55.4) mg/day in women and men, respectively. Mean (SD) reported intakes of betaine were 163 (51.5) mg/day in women and 173 (53.5) mg/day in men. Phosphatidylcholine was the most common choline form consumed and this accounted for 42% of total choline intake in women and 43% in men. Free choline and glycerophosphocholine accounted for 25 and 24% of the total choline intake, respectively, whereas sphingomyelin and phosphocholine each accounted for approximately 4–5%. Total choline intake was correlated with choline from all choline-containing compounds (*rho* = 0.79–0.89). In women, higher choline intake was associated with higher age, higher BMI, higher educational level, and higher levels of physical activity (Table 1). In men, higher choline intake was associated with higher BMI, higher educational level, and higher levels of physical activity (Table 2). Higher choline intake was associated with higher intake of protein as well as of B-vitamins and lower intake of carbohydrates in both women and men (Tables 1 and 2). In men, higher intake of choline was associated with lower intake of whole grains (Table 4). Baseline characteristics and daily dietary intakes in women and men by mortality status are given in Supplementary Table 1. Mortality cases were older at inclusion, had higher blood pressure and were more likely to be smokers, have lower educational level, and be less physically active than non-cases. In women, mortality cases had reported a higher intake of whole grains than non-cases.

**Table 1:** Baseline characteristics and diet of 52,246 women by quintiles of total estimated choline intake: the Västerbotten Intervention Programme

	Quintiles of total choline intake					P-trend
	1st *n* = 10,449	2nd *n* = 10,449	3rd *n* = 10,449	4th *n* = 10,449	5th *n* = 10,449	
Total estimated choline (mg/day)	197 (5.8)	231 (0.39)	251 (0.33)	273 (0.35)	325 (11.8)	
Age (year)	41.9 (1.2)	45.7 (0.53)	47.3 (1.1)	48.4 (1.6)	48.8 (2.2)	< 0.001
< 35 years	21.6	7.7	4.3	2.9	1.6	< 0.001
35–44 years	46.9	45.6	42.4	39.1	41.0	
45–54 years	23.7	29.4	30.0	30.0	26.3	
55–64 years	7.2	17.3	23.2	28.0	31.1	
Body mass index (kg/m^2^)	24.5 (0.73)	25.1 (0.61)	25.5 (0.60)	25.9 (0.71)	26.5 (0.66)	< 0.001
< 18.5	2.0	1.1	1.1	0.8	0.8	< 0.001
18.5–24.9	63.5	57.4	52.7	49.2	43.7	
25.0–29.9	15.6	28.9	32.0	34.1	34.4	
≥ 30.0	10.7	12.6	14.2	15.9	21.1	
Smoking						< 0.001
Current smoker	21.1	20.3	21.6	21.7	19.0	
Ex-smoker	24.9	27.7	27.8	28.7	31.3	
Non-smoker	53.2	21.0	49.8	48.5	48.9	
Missing value	0.9	0.9	0.9	1.0	0.8	
Educational level						< 0.001
Basic level, 9 years	27.0	33.3	36.8	38.0	32.6	
High school	39.5	33.4	28.7	27.0	28.4	
University	32.7	32.6	33.8	34.4	38.3	
Missing value	0.7	0.7	0.7	0.7	0.8	
Physical activity						< 0.001
Inactive	18.7	18.0	17.5	17.0	15.8	
Moderately inactive	31.4	32.2	31.9	30.3	27.3	
Moderately active	28.1	28.3	28.1	27.8	27.4	
Active	21.6	21.1	22.1	24.5	28.9	
Missing value	0.3	0.5	0.4	0.4	0.5	
Systolic blood pressure (mmHg)	119 (6.2)	122 (7.9)	124 (8.4)	125 (8.6)	125 (8.6)	< 0.001
Diastolic blood pressure (mmHg)	74 (3.1)	76 (3.3)	77 (3.4)	77 (3.4)	78 (3.2)	< 0.001
Serum cholesterol (mmol/l)	5.2 (0.44)	5.4 (0.52)	5.5 (0.53)	5.5 (0.54)	5.5 (0.53)	0.73
Fasting blood glucose (mmol/l)	5.3 (0.12)	5.4 (0.13)	5.4 (0.10)	5.4 (0.14)	5.5 (0.17)	< 0.001
Energy (kcal)	1,661 (163)	1,449 (124)	1,437 (128)	1,485 (125)	1,618 (123)	< 0.001
Carbohydrate (E%)	49.1 (1.9)	49.7 (2.3)	49.6 (2.5)	49.3 (2.9)	46.6 (4.3)	< 0.001
Fiber (g/1000 kcal)	10.7 (0.83)	11.6 (0.74)	11.9 (0.81)	12.1 (0.83)	12.0 (0.82)	< 0.001
Whole grain (g/1000 kcal)	38.8 (7.5)	41.4 (8.2)	41.1 (8.1)	40.6 (7.5)	36.3 (7.0)	< 0.001
Protein (E%)	12.9 (0.22)	14.3 (0.39)	15.1 (0.35)	16.0 (0.31)	17.7 (0.42)	< 0.001
Total fat (E%)	36.3 (2.0)	34.0 (2.0)	33.1 (2.2)	32.6 (2.5)	33.7 (3.8)	< 0.001
SFA (E%)	15.1 (0.58)	14.1 (0.62)	13.7 (0.72)	13.5 (0.90)	13.7 (1.5)	< 0.001
MUFA (E%)	11.5 (1.3)	11.4 (0.89)	11.3 (0.88)	11.2 (0.98)	11.8 (1.4)	< 0.001
PUFA (E%)	5.8 (0.61)	5.3 (0.56)	5.2 (0.54)	5.1 (0.60)	5.4 (0.74)	< 0.001
Betaine (mg/days)	155 (14.6)	164 (9.4)	165 (8.0)	166 (9.1)	165 (8.5)	0.51
Folate (µg/days)	195 (13.2)	220 (5.5)	235 (5.8)	251 (6.9)	289 (13.8)	< 0.001
Vitamin B-12 (µg/days)	3.8 (0.11)	4.5 (0.10)	4.9 (0.05)	5.4 (0.03)	6.3 (0.07)	< 0.001
Vitamin B-6 (mg/days)	1.4 (0.07)	1.6 (0.05)	1.6 (0.04)	1.7 (0.07)	1.8 (0.09)	< 0.001

Values represent percentages or means (SD). Mean values are adjusted for age and year of study participation. Comparison of baseline characteristics and diet by quintiles of choline intake were performed using linear regression (adjusted for age and year of study participant) for continuous data and Pearson Chi-square test for categorical data. Dietary intake was adjusted for total energy intake using nutrient density method (g/1000 kcal or E%). Total choline, betaine, vitamin B-6, vitamin B-12 and folate intakes were adjusted for total energy intake using the residual method

*E%* percent of total energy intake, *MUFA* monounsaturated fatty acid, *PUFA* polyunsaturated fatty acid, *SFA* saturated fatty acid

**Table 2:** Baseline characteristics and diet of 50,485 men by quintiles of total estimated choline intake: the Västerbotten Intervention Programme

	Quintiles of total choline intake					P-trend
	1st *n* = 10,097	2nd *n* = 10,097	3rd *n* = 10,097	4th *n* = 10,097	5th *n* = 10,097	
Total estimated choline (mg/days)	211 (3.8)	251 (0.25)	274 (0.04)	298 (0.39)	357 (8.6)	
Age (year)	46.0 (0.91)	47.0 (0.56)	47.0 (0.58)	46.6 (0.36)	46.0 (0.14)	0.66
< 35 years	18.9	7.7	5.4	3.6	2.4	< 0.001
35–44 years	29.6	38.6	42.3	47.2	53.3	
45–54 years	25.4	29.9	30.5	29.8	26.7	
55–64 years	26.1	23.8	21.8	19.4	17.6	
Body mass index (kg/m^2^)	25.8 (0.61)	26.2 (0.54)	26.4 (0.54)	26.6 (0.61)	27.1 (0.74)	< 0.001
< 18.5	0.5	0.3	0.3	0.2	0.2	< 0.001
18.5–24.9	45.9	40.3	37.2	36.1	31.5	
25.0–29.9	42.5	46.4	48.9	47.7	48.5	
≥ 30.0	11.1	13.0	13.6	15.9	19.7	
Smoking						0.003
Current smoker	19.2	18.6	18.4	19.1	18.3	
Ex-smoker	28.3	30.1	30.1	31.0	29.3	
Non-smoker	51.0	49.7	49.9	48.3	51.0	
Missing value	1.6	1.6	1.6	1.5	1.4	
Educational level						< 0.001
Basic level, 9 years	42.4	40.3	38.9	36.6	31.6	
High school	37.4	36.4	35.4	35.9	36.2	
University	19.7	22.8	25.2	27.1	31.6	
Missing value	0.6	0.6	0.5	0.4	0.6	
Physical activity						< 0.001
Inactive	18.4	18.9	18.3	18.6	16.9	
Moderately inactive	30.1	31.1	30.9	30.4	27.0	
Moderately active	29.6	28.8	28.5	28.7	27.8	
Active	21.5	20.9	22.0	22.1	28.0	
Missing value	0.4	0.2	0.3	0.3	0.3	
Systolic blood pressure (mmHg)	128 (5.7)	128 (5.5)	128 (5.3)	128 (5.1)	128 (4.7)	0.17
Diastolic blood pressure (mmHg)	80 (3.5)	80 (2.9)	81 (2.7)	80 (2.6)	80 (2.3)	0.04
Serum cholesterol (mmol/l)	5.5 (0.30)	5.6 (0.28)	5.6 (0.24)	5.6 (0.22)	5.5 (0.20)	< 0.001
Fasting blood glucose (mmol/l)	5.5 (0.21)	5.5 (0.20)	5.5 (0.23)	5.5 (0.23)	5.5 (0.30)	< 0.001
Energy (kcal)	2,239 (188)	1,896 (133)	1,854 (103)	1,919 (90)	2,156 (99)	< 0.001
Carbohydrate (E%)	46.8 (2.7)	46.3 (2.8)	45.9 (2.9)	45.3 (3.1)	42.6 (4.0)	< 0.001
Fiber (g/1000 kcal)	9.4 (1.0)	9.6 (1.1)	9.6 (1.0)	9.5 (0.96)	9.1 (0.94)	< 0.001
Whole grain (g/1000 kcal)	37.5 (2.1)	37.6 (1.8)	36.9 (2.1)	36.2 (2.7)	32.3 (3.3)	< 0.001
Protein (E%)	12.2 (0.17)	13.5 (0.10)	14.5 (0.20)	15.3 (0.24)	17.0 (0.44)	< 0.001
Total fat (E%)	38.4 (2.1)	37.0 (1.9)	36.4 (2.0)	36.2 (2.2)	37.5 (2.9)	< 0.001
SFA (E%)	16.5 (0.80)	15.7 (0.76)	15.3 (0.83)	15.1 (0.93)	15.4 (1.2)	< 0.001
MUFA (E%)	12.9 (1.2)	12.7 (1.1)	12.7 (1.1)	12.8 (1.1)	12.4 (1.2)	< 0.01
PUFA (E%)	5.9 (0.79)	5.8 (0.64)	5.7 (0.64)	5.7 (0.67)	6.0 (0.74)	< 0.001
Total fat (E%)	38.4 (2.1)	37.0 (1.9)	36.4 (2.0)	36.2 (2.2)	37.5 (2.9)	< 0.001
Betaine (mg/day)	166 (10.7)	174 (5.9)	176 (6.3)	177 (5.5)	173 (3.5)	< 0.001
Folate (µg/day)	204 (3.7)	224 (6.9)	234 (7.6)	245 (7.4)	260 (5.3)	< 0.001
Vitamin B-12 (µg/day)	4.7 (0.19)	5.6 (0.14)	6.2 (0.11)	6.7 (0.08)	7.9 (0.02)	< 0.001
Vitamin B-6 (mg/day)	1.8 (0.04)	2.0 (0.06)	2.1 (0.07)	2.2 (0.10)	2.4 (0.15)	< 0.001

Values represent percentages or means (SD). Mean values are adjusted for age and year of study participation. Comparison of baseline characteristics and diet by quintiles of choline intake were performed using linear regression (adjusted for age and year of study participant) for continuous data and Pearson Chi-square test for categorical data. Dietary intake was adjusted for total energy intake using nutrient density method (g/1000 kcal or E%). Total choline, betaine, vitamin B-6, vitamin B-12 and folate intakes were adjusted for total energy intake using the residual method

*E%* percent of total energy intake, *MUFA* monounsaturated fatty acid, *PUFA* polyunsaturated fatty acid, *SFA* saturated fatty acid

### Associations of choline and betaine intake on all-cause mortality

During a median follow-up of 15 y for women and 16 y for men, we identified 3,088 and 4,214 deaths in women and men, respectively. After adjusting for potential confounders, total choline intake was not associated with all-cause mortality, neither in women nor in men (Tables 3 and 4). Higher intake of betaine was associated with lower risk of all-cause mortality in women but not in men (Tables 3 and 4). In women, the HR (95% CI) for the highest compared to lowest betaine intake quintile was 0.86 (0.77, 0.97), *P*-trend < 0.01. Each 50-mg/day higher betaine intake was associated with a 5.3% reduction in the HR of all-cause mortality. In the analysis of choline intake from choline compounds and risk of all-cause mortality, intake of free choline was negatively associated with risk of all-cause mortality in women (Table 3). No other associations were found between intake of choline compounds and all-cause mortality in either sex (Tables 3 and 4). Analyses of choline as a continuous variable showed similar results with higher intake of choline from phosphatidylcholine being associated with increased risk for all-cause mortality in women (Table 3). Non-linear relationships with increased risk of all-cause mortality at lower intake of total choline for women and lower betaine intake for men were suggested (Supplemental Figs. 1 and 2).

**Table 3:** Hazard ratio (95% CIs) for all-cause mortality according to energy-adjusted estimated choline and betaine intake in 52,246 women: the Västerbotten Intervention Programme

	Q1	Q2	Q3	Q4	Q5	*P-*trend	Continuous	*P*
	*n* = 10,449	*n* = 10,449	*n* = 10,450	*n* = 10,449	*n* = 10,449			
Total choline	203 (188, 213)	231 (226, 237)	251 (246, 256)	272 (266, 279)	311 (297, 337)		Per 50 mg/dag	
No. of cases	403	607	699	734	645		3088	
Basic model	1.00 (ref)	1.00 (0.88, 1.13)	0.96 (0.84, 1.08)	0.94 (0.83, 1.07)	1.03 (0.91, 1.18)	0.89	1.04 (0.99, 1.09)	0.15
Adjusted model^a^	1.00 (ref)	1.00 (0.88, 1.14)	0.95 (0.84, 1.08)	0.93 (0.82, 1.05)	0.99 (0.87, 1.13)	0.55	1.01 (0.97, 1.06)	0.61
Phosphatidylcholine	76 (69, 81)	90 (87, 93)	100 (98, 103)	113 (109, 121)	143 (131,167)		Per 50 mg/dag	
No. of cases	544	659	766	702	417		3088	
Basic model	1.00 (ref)	0.89 (0.79, 1.00)	0.95 (0.85, 1.06)	0.94 (0.84, 1.05)	1.09 (0.96, 1.24)	0.21	1.10 (1.02. 1.19)	0.01
Adjusted^a^	1.00 (ref)	0.90 (0.80, 1.01)	0.94 (0.84, 1.06)	0.93 (0.83, 1.04)	1.06 (0.93, 1.20)	0.47	1.08 (1.00, 1.17	0.05
Sphingomyelin	9.7 (8.8, 10.3)	11.5 (11.1, 11.8)	12.8 (12.4, 13.1)	14.3 (13.8, 15.1)	17.4 (16.2, 19.7)		Per 5 mg/dag	
No. of cases	578	681	735	686	408		3088	
Basic model	1.00 (ref)	1.03 (0.92, 1.15)	1.08 (0.96, 1.20)	1.13 (1.01, 1.26)	1.19 (1.05, 1.35)	< 0.01	1.12 (1.05, 1.19)	< 0.001
Adjusted^a^	1.00 (ref)	1.02 (0.91, 1.14)	1.03 (0.92, 1.15)	1.07 (0.96, 1.20)	1.10 (0.96, 1.25)	0.09	1.06 (1.00, 1.14)	0.06
Phosphocholine	7.9 (6.8, 8.6)	10.0 (9.6, 10.4)	11.6 (11.2, 12.0)	13.3 (12.9, 13.8)	16.3 (15.2, 17.9)		Per 5 mg/dag	
No. of cases	434	551	655	718	730		3088	
Basic model	1.00 (ref)	0.94 (0.83, 1.07)	0.92 (0.81, 1.04)	0.95 (0.84, 1.08)	0.96 (0.85, 1.09)	0.84	1.01 (0.95, 1.06)	0.86
Adjusted^a^	1.00 (ref)	0.97 (0.85, 1.10)	0.95 (0.84, 1.08)	0.98 (0.87, 1.11)	0.97 (0.86, 1.09)	0.77	1.00 (0.94, 1.05)	0.85
Glycerophosphocholine	41.7 (36.4, 45.1)	52.4 (50.3, 54.4)	60.1 (58.2, 62.2)	69.1 (66.6, 72.0)	83.8 (79.0, 91.7)		Per 5 mg/dag	
No. of cases	389	472	644	742	841		3088	
Basic model	1.00 (ref)	0.83 (0.72, 0.95)	0.85 (0.75, 0.97)	0.86 (0.76, 0.98)	0.97 (0.86, 1.10)	0.40	1.01 (1.00, 1.02)	0.22
Adjusted^a^	1.00 (ref)	0.84 (0.73, 0.96)	0.86 (0.76, 0.98)	0.87 (0.77, 0.99)	0.93 (0.82, 1.05)	0.94	1.00 (0.99, 1.01)	1.00
Free choline	49.3 (44.6, 52.2)	57.9 (56.3, 59.5)	63.7 (62.3, 65.0)	69.4 (67.8, 71.1)	78.9 (75.6, 84.5)		Per 5 mg/dag	
No. of cases	298	490	675	753	872		3088	
Basic model	1.00 (ref)	0.89 (0.77, 1.03)	0.88 (0.77, 1.02)	0.79 (0.69, 0.91)	0.81 (0.71, 0.93)	< 0.001	0.98 (0.96, 1.00)	0.01
Adjusted^a^	1.00 (ref)	0.88 (0.76, 1.02)	0.87 (0.75, 1.00)	0.78 (0.67, 0.89)	0.81 (0.70, 0.93)	< 0.01	0.98 (0.96, 1.00)	0.01
Betaine	106 (90.3, 116)	136 (130, 142)	157 (152, 163)	181 (175, 189)	229 (210, 260)		Per 50 mg/dag	
No. of cases	634	693	637	595	529		3088	
Basic model	1.00 (ref)	0.93 (0.84, 1.04)	0.83 (0.74, 0.93)	0.78 (0.69, 0.87)	0.76 (0.68, 0.85)	< 0.001	0.90 (0.87, 0.94)	< 0.001
Adjusted^a^	1.00 (ref)	0.97 (0.87, 1.08)	0.90 (0.80, 1.00)	0.86 (0.77, 0.96)	0.86 (0.77, 0.97)	< 0.01	0.95 (0.91, 0.98)	< 0.01

Median (25th, 75th percentiles). Hazard ratios and 95% confidence intervals were calculated using Cox proportional hazard regression (time-dependent covariate: age) with quintile one as reference. Intake of choline and betaine is energy-adjusted using the residual method. *P* for trend was calculated with quintiles of choline and betaine intake as continuous variables in otherwise identical models

Basic model adjusted for age (continuous) and energy intake (continuous)

^a^Adjusted model adjusted for age (continuous), energy intake (continuous), BMI (continuous), smoking (categorical), educational level (categorical), and physical activity (categorical)

**Table 4:** Hazard ratio (95% CIs) for incidence all-cause mortality according to energy-adjusted estimated choline and betaine intake in 50,485 men: the Västerbotten Intervention Programme

	Q1	Q2	Q3	Q4	Q5	*P-*trend	Continuous	*P*
	*n* = 10,097	*n* = 10,097	*n* = 10,097	*n* = 10,097	*n* = 10,097			
Total choline	219 (201, 230)	252 (246, 257)	274 (269, 279)	298 (291, 305)	341 (326, 370)		Per 50 mg/dag	
No. of cases	986	913	882	779	654		4214	
Basic model	1.00 (ref)	0.98 (0.90, 1.08)	1.02 (0.93, 1.12)	1.00 (0.91, 1.10)	1.10 (1.00, 1.22)	0.10	1.02 (0.99, 1.06)	0.17
Adjusted^a^	1.00 (ref)	0.97 (0.89, 1.07)	1.01 (0.92, 1.11)	0.97 (0.88, 1.07)	1.06 (0.96, 1.17)	0.43	1.01 (0.98, 1.04)	0.54
Phosphatidylcholine	83.9 (74.5, 89.9)	102 (98.7, 106)	115 (112, 119)	132 (127, 137)	166 (153, 191)		Per 50 mg/dag	
No. of cases	1105	986	872	701	550		4214	
Basic model	1.00 (ref)	0.98 (0.90, 1.07)	0.95 (0.87, 1.04)	0.94 (0.85, 1.03)	1.09 (0.99, 1.21)	0.55	1.03 (0.98, 1.08)	0.31
Adjusted^a^	1.00 (ref)	0.97 (0.88, 1.06)	0.93 (0.85, 1.02)	0.90 (0.82, 0.99)	1.04 (0.94, 1.15)	0.61	1.00 (0.96, 1.05)	0.93
Sphingomyelin	10.1 (8.9, 10.9)	12.5 (12.0, 12.9)	14.1 (13.7, 14.5)	16.0 (15.4, 16.6)	19.8 (18.3, 22.4)		Per 5 mg/dag	
No. of cases	1217	1054	854	645	444		4214	
Basic model	1.00 (ref)	1.08 (0.99, 1.18)	1.07 (0.98, 1.17)	1.04 (0.95, 1.15)	1.09 (0.98, 1.22)	0.57	1.02 (0.99, 1.07)	0.17
Adjusted^a^	1.00 (ref)	1.04 (0.96, 1.13)	1.00 (0.92, 1.10)	0.96 (0.87, 1.06)	1.00 (0.89, 1.12)	0.50	0.99 (0.95, 1.04)	0.71
Phosphocholine	7.2 (6.1, 7.8)	9.2 (8.8, 9.6)	10.8 (10.4, 11.2)	12.5 (12.0, 13.0)	15.3 (14.3, 16.9)		Per 5 mg/dag	
No. of cases	866	774	800	880	894		4214	
Basic model	1.00 (ref)	0.89 (0.81, 0.98)	0.89 (0.81, 0.98)	0.97 (0.89, 1.07)	1.00 (0.91, 1.10)	0.43	1.01 (0.97, 1.06)	0.68
Adjusted^a^	1.00 (ref)	0.90 (0.81, 0.99)	0.92 (0.84, 1.02)	0.98 (0.90, 1.07)	1.00 (0.91, 1.10)	0.48	1.01 (0.96, 1.05)	0.73
Glycerophosphocholine	45.7 (40.2, 49.2)	56.5 (54.4, 58.6)	64.3 (62.4, 66.4)	73.4 (70.9, 76.8)	88.7 (84.0, 96.1)		Per 5 mg/dag	
No. of cases	775	839	780	933	887		4214	
Basic model	1.00 (ref)	1.01 (0.92, 1.12)	0.89 (0.81, 0.99)	1.05 (0.95, 1.15)	1.03 (0.93, 1.13)	0.38	1.01 (1.00, 1.01)	0.31
Adjusted^a^	1.00 (ref)	1.04 (0.94, 1.14)	0.92 (0.83, 1.01)	1.05 (0.96, 1.16)	1.02 (0.93, 1.12)	0.58	1.00 (0.99, 1.01)	0.50
Free choline	54.2 (49.7, 56.9)	62.5 (60.9, 63.9)	67.8 (66.6, 69.2)	73.5 (72.0, 75.3)	83.2 (79.8, 88.6)		Per 5 mg/dag	
No. of cases	738	786	787	895	1008		4214	
Basic model	1.00 (ref)	0.94 (0.85, 1.04)	0.88 (0.79, 0.97)	0.94 (0.86, 1.04)	1.02 (0.93, 1.13)	0.46	1.01 (1.00, 1.02)	0.21
Adjusted^a^	1.00 (ref)	0.95 (0.85, 1.05)	0.89 (0.80, 0.98)	0.94 (0.86, 1.04)	1.03 (0.93, 1.13)	0.46	1.01 (1.00, 1.02)	0.19
Betaine	115 (97.5, 126)	146 (140, 152)	168 (162, 173)	191 (185, 199)	239 (220, 271)		Per 50 mg/dag	
No. of cases	1134	952	784	692	652		4214	
Basic model	1.00 (ref)	0.95 (0.87, 1.04)	0.87 (0.79, 0.95)	0.85 (0.77, 0.93)	0.89 (0.81, 0.98)	< 0.001	0.96 (0.93, 0.99)	0.01
Adjusted^a^	1.00 (ref)	0.99 (0.91, 1.08)	0.92 (0.84, 1.01)	0.93 (0.84, 1.02)	0.99 (0.90, 1.09)	0.31	1.00 (0.97, 1.03)	0.83

Hazard ratios and 95% confidence intervals were calculated using Cox proportional hazard regression with quintile one as reference. Intake of choline and betaine is energy-adjusted using the residual method. *P* for trend was calculated with quintiles of choline and betaine intake as continuous variables in otherwise identical models

Basic model adjusted for age (continuous) and energy intake (continuous)

^a^Adjusted model adjusted for age (continuous), energy intake (continuous), BMI (continuous), smoking (categorical), educational level (categorical), and physical activity (categorical)

Interactions between choline and betaine intake and B-vitamin intake in their associations with all-cause mortality were explored and are reported in Supplemental Tables 2 and 3. Intake of vitamin B-12, vitamin B-6 or folate did not significantly modify the associations in women (*P*-interaction ≥ 0.25) (Supplemental Table 2). In men, intake of vitamin B-6 or vitamin B-12 did not significantly modify the associations (*P*-interaction ≥ 0.82) (Supplemental Table 3). However, in men with higher intake of folate, higher choline intake was associated with increased risk of all-cause mortality; HR (95% CI) for the highest compared to lowest quintile was 1.14 (1.00, 1.30), *P*-trend 0.03 (*P*-interaction 0.06).

To further explore the modifying effects of major dietary sources of choline and betaine, associations of total choline and betaine intake with all-cause mortality were stratified by median intake of animal protein and whole grain. (Supplemental Tables 4 and 5). Overall, level of animal protein or whole grain intake did not significantly modify the associations (*P*-interaction ≥ 0.21) (Supplemental Table 4). Analyses of associations between total choline, main sources of choline and betaine intake, and all-cause mortality by age groups are shown in Supplemental Tables 6 and 7. In women, total choline intake was not associated with all-cause mortality irrespective of age group (Supplemental Table 6). Choline intakes from phosphatidylcholine and sphingomyelin were associated with increased risk, whereas free choline intake was associated with lower risk of all-cause mortality in women ≥ 55 years. The interaction with age was significant only for phosphatidylcholine (*P* < 0.01) (Supplemental Table 6). In younger men (< 35–44 years), total choline as well as choline intakes from phosphocholine, glycerophosphocholine, and free choline were associated with an increased risk of all-cause mortality, but no statistically significant interactions of age group were found in men (Supplemental Table 7).

## Discussion

In this large population-based prospective cohort followed for an average of 16 years, we did not observe any associations between total choline intake and all-cause mortality, except for among young men. In women, intake of choline as free choline was associated with decreased risk of all-cause mortality. In men with a higher intake of folate, higher choline intake was associated with an increased risk of all-cause mortality. Higher betaine intake was associated with a lower risk of all-cause mortality in women but not in men.

Our findings of a null association between choline intake and all-cause mortality are to some extent in contrast to other studies. In three U.S. cohorts, total choline [11] and phosphatidylcholine [12] intakes were positively associated with all-cause mortality. Zheng et al. reported a higher phosphatidylcholine intake to be associated with increased risk of all-cause and CVD mortality among both women and men; however, they did not study intakes of total choline or other forms of choline [12]. Furthermore, in three ethnically diverse U.S and Chinese cohorts, higher intake of total choline was associated with increased risk of total mortality in blacks and Chinese but not in whites [13]. In the aforementioned study, various sources of choline intake were studied, and some contrasting cohort-specific findings were reported. For example, intake of choline from phosphatidylcholine was positively associated with total mortality in the U.S. cohort but not in the Chinese cohort. We found no material difference in results for the different types of choline sources apart from a negative association between free choline intake and all-cause mortality in women; this is in contrast with results from Yang et al. who observed either a positive or null association between free choline intake and all-cause mortality [13]. Thus, intake of choline from different choline-containing compounds may have different associations with mortality and this should be considered in future studies. In the previous studies, positive associations were generally stronger in individuals with obesity [12] or diabetes [12, 13]. The range of choline and betaine intake in the current cohort was limited and may impact possibilities to detect associations; however, the range is in line with previous studies [11]. Furthermore, genetic variants related to choline metabolism and function have been shown to alter dietary requirements of choline and these genetic variants differ between populations [29]. Overall, habitual dietary habits, gut microbiota composition, genetic variation, and morbidity status may explain different results between populations. The reason why we found associations between higher intakes of total choline as well as individual forms of choline and increased mortality among young men is intriguing and difficult to explain. It may be a chance finding.

Epidemiological studies on betaine intake and mortality are limited. In contrast to our result of a lower risk of all-cause mortality in women with a higher intake of betaine, intake of betaine was positively associated with total mortality in the Chinese cohort and a null association was reported in the U.S. cohorts in the study by Yang et al. [13]. In a small (*n* = 1292) Chinese cohort of adults with coronary artery disease, betaine intake was associated with decreased risk of cardiovascular mortality but not all-cause mortality [30]. Rich sources of betaine are plant-based foods such as cereals and cereal products [5]. Higher intake of betaine may be related to concurrent higher intake of whole grain explaining negative associations with all-cause mortality. Suggestive of this, in our study, the negative association between betaine intake and all-cause mortality was stronger in women with a higher intake of whole grains.

The underlying mechanisms of any health-disease relationship are elusive. Concurrently, dietary choline and betaine have been hypothesized to have both beneficial and harmful effects. A concern of high choline intake has been the gut microbiota-dependent metabolization of TMAO, which has been associated with increased risk of CVD incidence, CVD mortality, and all-cause mortality in prospective cohort studies [9, 10]. However, whether elevated TMAO is a cause or consequence of CVD is unclear. Concentrations of TMAO are influenced not only by diet, but also by gut microbiome, enzyme activity, and kidney function [31].

Elevated systemic concentrations of total Hcy have been associated with CVD [1]. Higher intakes of choline and betaine promote Hcy remethylation to methionine and have been associated with decreased circulating concentrations of Hcy [6, 7]. The causal link of Hcy to CVD is, however, uncertain. In a meta-analysis of eight randomized controlled trials, lowering Hcy by B-vitamin supplementation had no effect on cardiovascular events, cancer incidence or all-cause, cancer, or CVD mortality [32]. Choline and betaine may modulate DNA methylation by affecting availability of the universal methyl donor S-adenosylmethionine, converted from Hcy via methionine [33]. Methylation may be involved in carcinogenesis and atherogenic processes [34]. Furthermore, a beneficial effect of higher intake of choline and betaine may be decreased low-grade inflammation. Higher intakes of choline and betaine have been associated with decreased circulating concentrations of inflammatory markers, such as C-reactive protein, interleukin-6, and tumor necrosis factor alpha [8].

The metabolisms of choline, betaine, folate, and B-vitamins are interrelated, and it is recognized that in the presence of folate or vitamin B-12 deficiency, choline and betaine requirements are increased [1]. Therefore, interrelationships of B-vitamins and choline and betaine intake should be considered when assessing diet–health relationships of choline and betaine intake. However, we did not find any material differences in results when exploring interactions between choline or betaine intake with vitamin B-12, vitamin B-6, or folate intake in women. In men with higher intake of folate, higher total choline intake was associated with an increased risk of all-cause mortality. This may be an overall effect of higher intakes of methyl-group nutrients, possibly leading to alterations in DNA methylation. However, it may also be a chance finding.

The main strength of the current study is the population-based VIP cohort where selection bias seems small [19] and which includes a large sample size, reliable mortality data as well as standardized, validated, and consistent methods of dietary and lifestyle assessments. There are some limitations that merit attention. Even though adjustment for confounding has been done, the risk of residual confounding cannot be excluded. Moreover, it is challenging to measure true dietary intake. Intake of choline has not been validated in the current FFQ nor from dietary assessments in other cohorts. To date, no good dietary biomarkers of choline intake have been identified, making objective validation difficult. Currently, there is no Swedish dietary database of choline and betaine content of foods, and the only available database is the USDA database. There will be differences in content of choline and betaine in foods between countries due to animal feeding and cultivation as well as differences in cooking methods, recipes of mixed dishes, and lack of cultural foods. It is crucial that national databases are created to improve our present knowledge of the effects of dietary choline and betaine in different populations. Eggs are high in choline, specifically phosphatidylcholine, and the early versions of the FFQ in VIP did not include a separate question of egg intake, only egg as part of dishes. Even though we imputed the median intake of choline from egg from the later FFQs, we cannot exclude individual under- or overestimations of choline from egg consumption (whole egg and egg dishes), affecting our ability to capture variations in choline intake.

In conclusion, the present results suggest that total choline intake is not an overall predictor of all-cause mortality in women and men. Associations of choline intake with all-cause mortality may be modified by age or folate intake and differently so depending on sex and should be explored further. Higher betaine intake was suggestive to be beneficial in relation to all-cause mortality in women.

### Supplementary Information

Below is the link to the electronic supplementary material.

Supplementary file1 (PDF 302 KB)

## Data Availability

Data cannot be made freely available as they are subject to secrecy in accordance with the Swedish Public Access to Information and Secrecy Act [Offentlighets-och sekretesslagen, OSL, 2009:400], but can be made available to researchers upon request (subject to a review of secrecy). Requests for data should be made to Anna Winkvist, anna.winkvist@umu.se, or directed to Ingvar Bergdahl, director of the Biobank Research Unit, Umeå University, Sweden, ebf@umu.se.

## Abbreviations

BMI: Body mass index
CVD: Cardiovascular disease
FFQ: Food-frequency questionnaire
FIL: Food intake level
Hcy: Homocysteine
NSDD: Northern Sweden Diet Database
TMA: Trimethylamine
TMAO: Trimethylamine-*N*-oxide
USDA: U.S. Department of Agriculture
VIP: Västerbotten Intervention Programme
