# Supplementary material for: Associations of dietary choline and betaine with all-cause mortality: a prospective study in a large Swedish cohort
Source: Eur J Nutr. 2024 Jan 4;63(3):785–96. doi: 10.1007/s00394-023-03300-y (PMC10948568; doi:10.1007/s00394-023-03300-y)
Supplement: Supplementary file 1 — Supplementary file1 (PDF 302 KB) [file 394_2023_3300_MOESM1_ESM.pdf]

Associations of dietary choline and betaine with all-cause mortality: a prospective study in a large Swedish cohort

European Journal of Nutrition

Therese Karlsson[1,2], Anna Winkvist[1,3], Anna Strid[1], Bernt Lindahl[3], Ingegerd Johansson[4]

[1]Department of Internal Medicine and Clinical Nutrition, Institute of Medicine, Sahlgrenska Academy, University of Gothenburg, Gothenburg, Sweden. therese.karlsson@gu.se

[2]Department of Life Sciences, Division of Food and Nutrition Science, Chalmers University of Technology, Gothenburg, Sweden

[3]Department of Public Health and Clinical Medicine, Sustainable Health, Umeå University, Umeå, Sweden

[4]Department of Odontology, Umeå University, Umeå, Sweden

Supplemental Table 1. Baseline characteristics of 102,731 men and women in the Västerbotten Intervention Programme.

	**Women**		**Men**	
	Non-cases *n*=49,158	Cases *n*=3,088	Non-cases *n*=46,271	Cases *n*=4,214
Age (y)	45.9 (0.16)	54.7 (0.17)	45.8 (0.35)	54.6 (0.46)
< 35 y	8.0	1.6	8.1	1.5
35-44 y	45.1	10.6	45.1	10.2
45-54 y	27.9	27.5	28.4	29.4
55-64 y	19.0	60.3	18.4	58.9
BMI (kg/m^2)	25.4 (0.81)	26.4 (0.60)	26.4 (0.70)	26.7 (0.37)
<18.5	1.1	1.7	0.3	0.5
18.8-24.9	53.9	43.9	38.6	34.4
25.0-29.9	30.4	34.3	46.7	48.2
≥30.0	14.6	20.1	14.5	17.0
Smoking				
Current smoker	20.0	33.1	17.6	31.1
Ex-smoker	28.3	24.3	29.4	34.2
Non-smoker	50.9	41.5	51.5	32.9
Missing value	0.9	1.1	1.5	1.8
Educational level				
Basic level, 9 years	31.3	69.1	35.1	69.4
High school	32.5	14.0	38.1	15.7
University	35.5	15.4	26.3	13.9
Missing value	0.7	1.5	0.5	1.1
Physical activity				
Inactive	17.1	21.5	18.0	21.0
Moderately inactive	30.3	36.0	29.4	35.5
Moderately active	28.0	26.3	28.7	28.0
Active	24.2	15.0	23.7	14.6
Missing value	0.4	1.2	0.3	0.9
Systolic blood pressure (mmHg)	122 (7.8)	135 (7.6)	127 (4.8)	136 (4.6)
Diastolic blood pressure (mmHg)	76 (3.3)	81 (2.9)	80 (2.7)	84 (1.6)
S-Cholesterol (mmol/l)	5.4 (0.50)	6.1 (0.45)	5.5 (0.23)	5.9 (0.21)
Fasting blood glucose (mmol/l)	5.4 (0.14)	5.6 (0.17)	5.5 (0.21)	5.8 (0.21)
Energy (kcal)	1535 (131)	1446 (107)	2011 (129)	2037 (61)
Carbohydrate (E%)	48.7 (2.9)	51.2 (1.4)	45.1 (3.3)	48.3 (1.7)
Fiber (g/1000 kcal)	11.6 (0.92)	12.2 (0.64)	9.4 (1.0)	10.4 (0.69)
Whole grain (g/1000 kcal)	39.1 (7.3)	48.0 (7.2)	36.0 (2.5)	37.1 (0.92)

Protein (E%)	15.2 (0.59)	15.2 (0.24)	14.5 (0.44)	14.2 (0.09)
Total fat (E%)	34.1 (2.7)	32.0 (1.3)	37.3 (2.2)	35.2 (1.3)
SFA (E%)	14.0 (0.96)	13.5 (0.38)	15.6 (0.89)	15.0 (0.33)
MUFA (E%)	11.5 (1.1)	10.8 (0.38)	13.0 (1.1)	11.6 (1.1)
PUFA (E%)	5.4 (0.61)	4.7 (0.44)	5.9 (0.66)	5.0 (0.60)
Choline intake (mg/d)	255 (94)	260 (42)	279 (56)	271 (51)
Betaine intake (mg/d)	163 (10.8)	160 (9.4)	174 (5.4)	164 (8.1)
Folate (μg/d)	238 (18.5)	233 (11.2)	234 (5.0)	231 (5.6)
Vitamin B-12 (μg/d)	4.9 (0.30)	5.2 (0.37)	6.2 (0.26)	6.1 (0.10)
Vitamin B-6 (mg/d)	1.6 (0.08)	1.7 (0.06)	2.1 (0.05)	2.0 (0.10)

Values represent percentages or means (SD). Mean values are adjusted for age and year of study participation. Dietary intake was adjusted for total energy intake using nutrient density method (g/1000 kcal or E%).

Missing data: Diastolic BP (*n*=778), cholesterol (*n*=383), education (*n*=652), glucose (*n*=446), smoking (*n*=1240), systolic BP (*n*=722). E%, percent of total energy intake; MUFA, monounsaturated fatty acid; PUFA, polyunsaturated fatty acid; SFA, saturated fatty acid.

Supplemental Table 2. Hazard ratio (95% CIs) for all-cause mortality per quintile of energy-adjusted estimated total choline intake in 52,246 women in the Västerbotten Intervention Programme by median intake of folate, vitamin B-12 and vitamin B-6

	**1**	**2**	**3**	**4**	**5**	***P* trend**	***P* interaction**
	n = 10,449	*n* = 10,449	*n* = 10,450	*n* = 10,449	*n* = 10,449		
Total choline intake							0.25
≤ median folate intake[1]	1.00 (ref)	1.05 (0.90, 1.24)	1.05 (0.90, 1.23)	0.93 (0.79, 1.10)	1.10 (0.91, 1.32)	0.90	
> median folate intake[1]	1.00 (ref)	0.93 (0.75, 1.15)	0.80 (0.65, 0.99)	0.94 (0.77, 1.15)	0.93 (0.77, 1.13)	0.94	
Total choline intake							0.45
≤ median vitamin B-12 intake[1]	1.00 (ref)	1.02 (0.88, 1.19)	0.97 (0.83, 1.13)	0.89 (0.75, 1.05)	0.90 (0.70, 1.15)	0.08	
> median vitamin B-12 intake[1]	1.00 (ref)	0.94 (0.73, 1.20)	0.90 (0.71, 1.14)	0 92 (0.73, 1.15)	0.97 (0.78, 1.21)	0.78	
Total choline intake							0.39
≤ median vitamin B-6 intake[1]	1.00 (ref)	0.99 (0.84, 1.17)	0.96 (0.81, 1.13)	0.88 (0.74, 1.05)	0.99 (0.80, 1.20)	0.29	
> median vitamin B-6 intake[1]	1.00 (ref)	1.01 (0.82, 1.24)	0.92 (0.75, 1.12)	1.00 (0.83, 1.20)	1.02 (0.85, 1.23)	0.68	

Hazard ratios and 95% confidence intervals were calculated using Cox proportional hazards regression (time-dependent covariate: age) with quintile one as reference. Intake of choline is energy-adjusted using the residual method. *P* for trend was calculated with quintiles of choline intake as continuous variables in otherwise identical models.

[1]Model adjusted for age (continuous), energy intake (continuous), BMI (continuous), smoking (categorical), educational level (categorical) and physical activity (categorical).

Supplemental Table 3. Hazard ratio (95% CIs) for all-cause mortality per quintile of energy-adjusted estimated total choline intake in 50,485 men in the Västerbotten Intervention Programme by median intake of folate, vitamin B-12 and vitamin B-6

	**1**	**2**	**3**	**4**	**5**	***P* trend**	**P interaction**
	n = 10,097	*n* = 10,097	*n* = 10,097	*n* = 10,097	*n* = 10,097		
Total choline intake							0.06
≤ median folate intake[1]	1.00 (ref)	0.94 (0.83, 1.07)	1.10 (0.88, 1.14)	0.90 (0.79, 1.04)	0.98 (0.83, 1.16)	0.49	
> median folate intake[1]	1.00 (ref)	1.01 (0.88, 1.16)	1.03 (0.90, 1.19)	1.08 (0.94, 1.24)	1.14 (1.00, 1.30)	0.03	
Total choline intake							0.82
≤ median vitamin B12 intake[1]	1.00 (ref)	0.96 (0.85, 1.07)	0.99 (0.87, 1.11)	0.96 (0.83, 1.10)	0.94 (0.75, 1.17)	0.57	
> median vitamin B12 intake[1]	1.00 (ref)	0.96 (0.81, 1.12)	0.99 (0.85, 1.16)	0.95 (0.82, 1.11)	1.06 (0.92, 1.23)	0.34	
Total choline intake							0.85
≤ median vitamin B6 intake[1]	1.00 (ref)	0.94 (0.83, 1.06)	1.01 (0.89, 1.15)	0.93 (0.81, 1.07)	1.05 (0.87, 1.26)	1.00	
> median vitamin B6 intake[1]	1.00 (ref)	1.00 (0.86, 1.15)	0.98 (0.85, 1.14)	1.00 (0.87, 1.15)	1.06 (0.92, 1.21)	0.43	

Hazard ratios and 95% confidence intervals were calculated using Cox proportional hazards regression with quintile one as reference. Intake of choline is energy-adjusted using the residual method. *P* for trend was calculated with quintiles of choline intake as continuous variables in otherwise identical models.

[1]Model adjusted for age (continuous), energy intake (continuous), BMI (continuous), smoking (categorical), educational level (categorical) and physical activity (categorical).

Supplemental Table 4. Hazard ratio (95% CIs) for all-cause mortality per quintile of energy-adjusted estimated total choline and betaine intake in 52,246 women in the Västerbotten Intervention Programme by median intake of animal protein and whole grain

	**1**	**2**	**3**	**4**	**5**	***P* trend**	***P* interaction**
	n = 10,449	*n* = 10,449	*n* = 10,450	*n* = 10,449	*n* = 10,449		
Total choline intake							0.26
≤ median animal protein intake[1]	1.00 (ref)	0.96 (0.83, 1.12)	0.94 (0.80, 1.09)	0.87 (0.74, 1.02)	0.99 (0.80, 1.23)	0.23	
> median animal protein intake[1]	1.00 (ref)	1.11 (0.86, 1.43)	0.98 (0.77, 1.26)	1.05 (0.83, 1.32)	1.05 (0.83, 1.32)	0.88	
Betaine intake							0.21
≤ median whole grain intake[1]	1.00 (ref)	0.95 (0.82, 1.11)	0.84 (0.71, 1.00)	0.82 (0.68, 1.00)	0.94 (0.73, 1.21)	0.06	
> median whole grain intake[1]	1.00 (ref)	0.95 (0.81, 1.12)	0.92 (0.78, 1.08)	0.87 (0.74, 1.02)	0.84 (0.73, 0.98)	0.01	

Hazard ratios and 95% confidence intervals were calculated using Cox proportional hazards regression (time-dependent covariate: age) with quintile one as reference. Intake of choline is energy-adjusted using the residual method. *P* for trend was calculated with quintiles of choline intake as continuous variables in otherwise identical models.

[1]Model adjusted for age (continuous), energy intake (continuous), BMI (continuous), smoking (categorical), educational level (categorical) and physical activity (categorical).

Supplemental Table 5. Hazard ratio (95% CIs) for all-cause mortality per quintile of energy-adjusted estimated total choline and betaine intake in 50,485 men in the Västerbotten Intervention Programme by median intake of animal protein and whole grain

	**1**	**2**	**3**	**4**	**5**	***P* trend**	***P* interaction**
	n = 10,097	*n* = 10,097	*n* = 10,097	*n* = 10,097	*n* = 10,097		
Total choline intake							0.45
≤ median animal protein intake[1]	1.00 (ref)	0.95 (0.85, 1.07)	0.96 (0.85, 1.09)	0.97 (0.84, 1.11)	1.18 (0.96, 1.45)	0.60	
> median animal protein intake[1]	1.00 (ref)	0.98 (0.83, 1.15)	1.07 (0.92, 1.24)	0.99 (0.85, 1.15)	1.07 (0.93, 1.24)	0.34	
Betaine intake							0.21
≤ median whole grain intake[1]	1.00 (ref)	1.06 (0.93, 1.19)	0.93 (0.82, 1.07)	0.96 (0.83, 1.12)	1.16 (0.96, 1.39)	0.92	
> median whole grain intake[1]	1.00 (ref)	0.91 (0.80, 1.04)	0.90 (0.78, 1.03)	0.90 (0.79, 1.03)	0.92 (0.81, 1.05)	0.25	

Hazard ratios and 95% confidence intervals were calculated using Cox proportional hazards regression with quintile one as reference. Intake of choline is energy-adjusted using the residual method. *P* for trend was calculated with quintiles of choline intake as continuous variables in otherwise identical models.

[1]Model adjusted for age (continuous), energy intake (continuous), BMI (continuous), smoking (categorical), educational level (categorical) and physical activity (categorical).

Supplemental Table 6. Hazard ratio (95% CIs) for all-cause mortality per quintile of energy-adjusted estimated choline and betaine intake in 52,246 women in the Västerbotten Intervention Programme by age group.

		**1**	**2**	**3**	**4**	**5**	***P* trend**	***P* interaction**
	N/Cases							
Total choline intake[1]								0.07
<35-44 y	26,454/378	1.00 (ref)	0.82 (0.62, 1.09)	1.04 (0.79, 1.38)	0.77 (0.55, 1.06)	0.82 (0.57, 1.19)	0.23	
45-54 y	14,576/848	1.00 (ref)	1.21 (0.96, 1.51)	1.11 (0.88, 1.39)	1.16 (0.93, 1.46)	1.18 (0.92, 1.50)	0.34	
≥55 y	11,216/1,862	1.00 (ref)	1.03 (0.85, 1.25)	0.93 (0.77, 1.12)	0.93 (0.77, 1.12)	1.01 (0.84, 1.22)	0.77	
Phosphatidylcholine intake[1]								<0.01
<35-44 y	26,454/378	1.00 (ref)	0.92 (0.68, 1.23)	0.98 (0.73, 1.31)	0.75 (0.54, 1.03)	0.83 (0.58, 1.18)	0.12	
45-54 y	14,576/848	1.00 (ref)	0.96 (0.78, 1.19)	0.97 (0.78, 1.20)	1.04 (0.84, 1.29)	0.97 (0.75, 1.25)	0.84	
≥55 y	11,216/1,862	1.00 (ref)	0.89 (0.76, 1.04)	0.95 (0.82, 1.11)	0.94 (0.81, 1.10)	1.20 (1.02, 1.43)	0.04	
Sphingomyelin intake[1]								0.83
<35-44 y	26,454/378	1.00 (ref)	0.82 (0.61, 1.11)	0.96 (0.71, 1.29)	0.80 (0.59, 1.10)	0.76 (0.53, 1.08)	0.13	
45-54 y	14,576/848	1.00 (ref)	1.09 (0.88, 1.34)	1.19 (0.97, 1.46)	1.15 (0.92, 1.42)	1.14 (0.89, 1.45)	0.19	
≥55 y	11,216/1,862	1.00 (ref)	1.05 (0.91, 1.22)	1.01 (0.87, 1.17)	1.13 (0.97, 1.30)	1.21 (1.02, 1.43)	0.02	
Phosphocholine intake[1]								0.82
<35-44 y	26,454/378	1.00 (ref)	0.80 (0.59, 1.07)	0.76 (0.55, 1.04)	0.95 (0.71, 1.29)	1.03 (0.76, 1.40)	0.83	
45-54 y	14,576/848	1.00 (ref)	1.11 (0.89, 1.39)	1.15 (0.92, 1.44)	1.14 (0.91, 1.43)	1.13 (0.90, 1.42)	0.31	
≥55 y	11,216/1,862	1.00 (ref)	0.97 (0.80, 1.17)	0.92 (0.77, 1.10)	0.94 (0.79, 1.12)	0.91 (0.77, 1.09)	0.33	
Glycerophosphocholine intake[1]								0.33
<35-44 y	26,454/378	1.00 (ref)	0.63 (0.46, 0.86)	0.76 (0.56, 1.03)	0.90 (0.67, 1.21)	0.76 (0.55, 1.04)	0.30	
45-54 y	14,576/848	1.00 (ref)	1.09 (0.86, 1.38)	1.16 (0.92, 1.46)	1.03 (0.81, 1.30)	1.23 (0.98, 1.54)	0.17	
≥55 y	11,216/1,862	1.00 (ref)	0.80 (0.65, 0.99)	0.77 (0.63, 0.93)	0.80 (0.67, 0.97)	0.85 (0.71, 1.02)	0.69	
Free choline intake[1]								0.41
<35-44 y	26,454/378	1.00 (ref)	1.01 (0.76, 1.35)	1.18 (0.88, 1.58)	0.98 (0.71, 1.35)	0.95 (0.67, 1.35)	0.90	
45-54 y	14,576/848	1.00 (ref)	0.95 (0.74, 1.23)	0.96 (0.75, 1.23)	0.84 (0.65, 1.07)	0.90 (0.70, 1.15)	0.21	
≥55 y	11,216/1,862	1.00 (ref)	0.84 (0.67, 1.07)	0.80 (0.64, 0.99)	0.73 (0.59, 0.91)	0.76 (0.62, 0.95)	0.01	
Betaine intake[1]								0.36
<35-44 y	26,454/378	1.00 (ref)	1.26 (0.95, 1.68)	1.00 (0.72, 1.38)	1.18 (0.85, 1.63)	1.39 (1.01, 1.92)	0.12	
45-54 y	14,576/848	1.00 (ref)	0.87 (0.71, 1.06)	0.90 (0.73, 1.10)	0.74 (0.60, 0.93)	0.82 (0.66, 1.03)	0.02	

≥55 y	11,216/1,862	1.00 (ref)	0.98 (0.85, 1.14)	0.90 (0.77, 1.04)	0.88 (0.76, 1.02)	0.83 (0.71, 0.97)	<0.01	

Hazard ratios and 95% confidence intervals were calculated using Cox proportional hazards regression with quintile one as reference. Intake of choline is energy-adjusted using the residual method. *P* for trend was calculated with quintiles of choline intake as continuous variables in otherwise identical models.

[1]Model adjusted for energy intake (continuous), BMI (continuous), smoking (categorical), educational level (categorical) and physical activity (categorical).

Supplemental Table 7. Hazard ratio (95% CIs) for all-cause mortality per quintile of energy-adjusted estimated choline and betaine intake in 50,485 men in the Västerbotten Intervention Programme by age group.

		**1**	**2**	**3**	**4**	**5**	***P* trend**	***P* interaction**
	N/Cases							
Total choline intake[1]								0.12
<35-44 y	25,135/494	1.00 (ref)	1.17 (0.85, 1.60)	1.25 (0.92, 1.70)	1.34 (1.00, 1.80)	1.69 (1.28, 2.24)	<0.001	
45-54 y	14,370/1,238	1.00 (ref)	1.03 (0.86, 1.24)	1.00 (0.83, 1.20)	1.11 (0.92, 1.33)	1.05 (0.86, 1.27)	0.41	
≥55 y	10,980/2,482	1.00 (ref)	0.96 (0.86, 1.07)	1.05 (0.93, 1.17)	0.92 (0.81, 1.04)	1.06 (0.93, 1.22)	0.76	
Phosphatidylcholine intake[1]								0.42
<35-44 y	25,135/494	1.00 (ref)	0.87 (0.64, 1.18)	1.06 (0.79, 1.42)	1.02 (0.77, 1.36)	1.24 (0.93, 1.64)	0.86	
45-54 y	14,370/1,238	1.00 (ref)	1.04 (0.87, 1.23)	0.91 (0.76, 1.09)	0.89 (0.74, 1.06)	1.11 (0.91, 1.35)	0.84	
≥55 y	10,980/2,482	1.00 (ref)	0.97 (0.87, 1.08)	0.96 (0.85, 1.08)	0.94 (0.82, 1.07)	1.02 (0.89, 1.18)	0.79	
Sphingomyelin intake[1]								0.90
<35-44 y	25,135/494	1.00 (ref)	1.07 (0.79, 1.46)	1.13 (0.83, 1.53)	1.10 (0.82, 1.49)	1.27 (0.95, 1.70)	0.13	
45-54 y	14,370/1,238	1.00 (ref)	0.99 (0.84, 1.17)	0.93 (0.79, 1.11)	0.92 (0.77, 1.10)	0.93 (0.76, 1.14)	0.25	
≥55 y	10,980/2,482	1.00 (ref)	1.08 (0.97, 1.20)	1.06 (0.94, 1.19)	1.01 (0.89, 1.16)	1.03 (0.88, 1.21)	0.71	
Phosphocholine intake[1]								0.84
<35-44 y	25,135/494	1.00 (ref)	0.90 (0.66, 1.22)	1.24 (0.93, 1.64)	1.21 (0.92, 1.61)	1.32 (1.01, 1.73)	<0.01	
45-54 y	14,370/1,238	1.00 (ref)	0.80 (0.67, 0.95)	0.87 (0.73, 1.04)	0.87 (0.73, 1.04)	0.92 (0.77, 1.09)	0.65	
≥55 y	10,980/2,482	1.00 (ref)	0.96 (0.85, 1.09)	0.92 (0.81, 1.04)	1.03 (0.91, 1.17)	1.03 (0.91, 1.17)	0.32	
Glycerophosphocholine intake[1]								0.29
<35-44 y	25,135/494	1.00 (ref)	1.08 (0.80, 1.47)	1.10 (0.81, 1.48)	1.38 (1.04, 1.83)	1.40 (1.07, 1.84)	<0.01	
45-54 y	14,370/1,238	1.00 (ref)	0.99 (0.82, 1.19)	0.88 (0.73, 1.06)	0.97 (0.81, 1.16)	1.01 (0.85, 1.21)	0.95	
≥55 y	10,980/2,482	1.00 (ref)	1.07 (0.94, 1.21)	0.93 (0.82, 1.06)	1.09 (0.96, 1.23)	1.02 (0.89, 1.16)	0.70	
Free choline intake[1]								0.23
<35-44 y	25,135/494	1.00 (ref)	1.23 (0.90, 1.68)	1.21 (0.89, 1.67)	1.39 (1.03, 1.87)	1.72 (1.32, 2.26)	<0.001	
45-54 y	14,370/1,238	1.00 (ref)	0.88 (0.72, 1.08)	0.81 (0.66, 0.99)	0.93 (0.77, 1.13)	1.03 (0.85, 1.23)	0.29	
≥55 y	10,980/2,482	1.00 (ref)	0.97 (0.86, 1.10)	0.92 (0.81, 1.05)	0.95 (0.84, 1.08)	1.01 (0.89, 1.15)	0.96	
Betaine intake[1]								0.67
<35-44 y	25,135/494	1.00 (ref)	0.98 (0.74, 1.31)	1.08 (0.82, 1.44)	1.13 (0.85, 1.50)	1.21 (0.92, 1.59)	0.11	
45-54 y	14,370/1,238	1.00 (ref)	0.93 (0.78, 1.10)	0.96 (0.80, 1.14)	0.90 (0.75, 1.08)	1.00 (0.83, 1.19)	0.81	

≥55 y	10,980/2,482	1.00 (ref)	1.05 (0.94, 1.17)	0.90 (0.80, 1.02)	0.95 (0.83, 1.07)	1.00 (0.88, 1.14)	0.36	

Hazard ratios and 95% confidence intervals were calculated using Cox proportional hazards regression with quintile one as reference. Intake of choline is energy-adjusted using the residual method. *P* for trend was calculated with quintiles of choline intake as continuous variables in otherwise identical models.

[1]Model adjusted for energy intake (continuous), BMI (continuous), smoking (categorical), educational level (categorical) and physical activity (categorical).

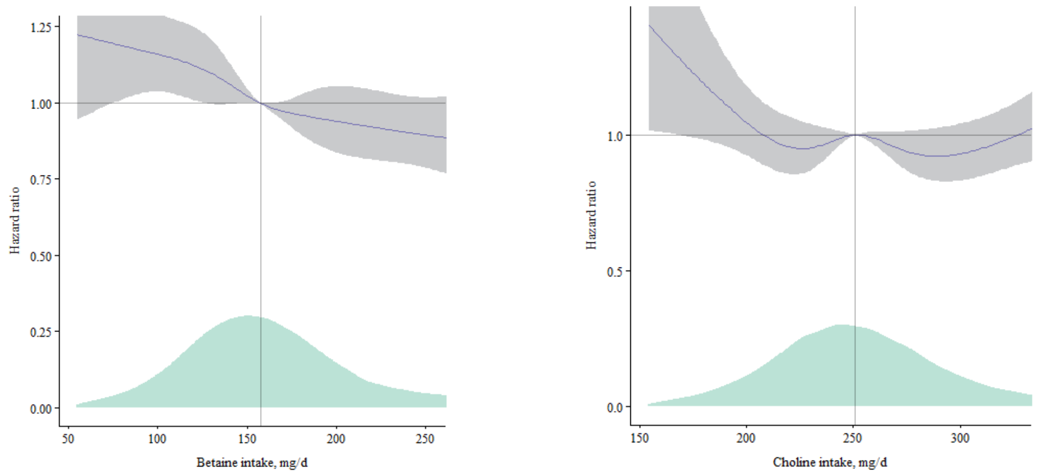

Supplemental Figure 1. Relation of betaine and total choline intake with risk of all-cause mortality in women. The solid lines show the observed association (hazard ratios) and the shaded areas 95% confidence intervals. Density plots indicate the distribution of choline and betaine intakes. The median was set as the reference. Model adjusted for energy intake (continuous), BMI (continuous), smoking (categorical), educational level (categorical) and physical activity (categorical). Choline and betaine intake is energy-adjusted using the residual method. Participants with intake the <1st and >99th percentiles were excluded from the analysis to minimize effects of extreme values.

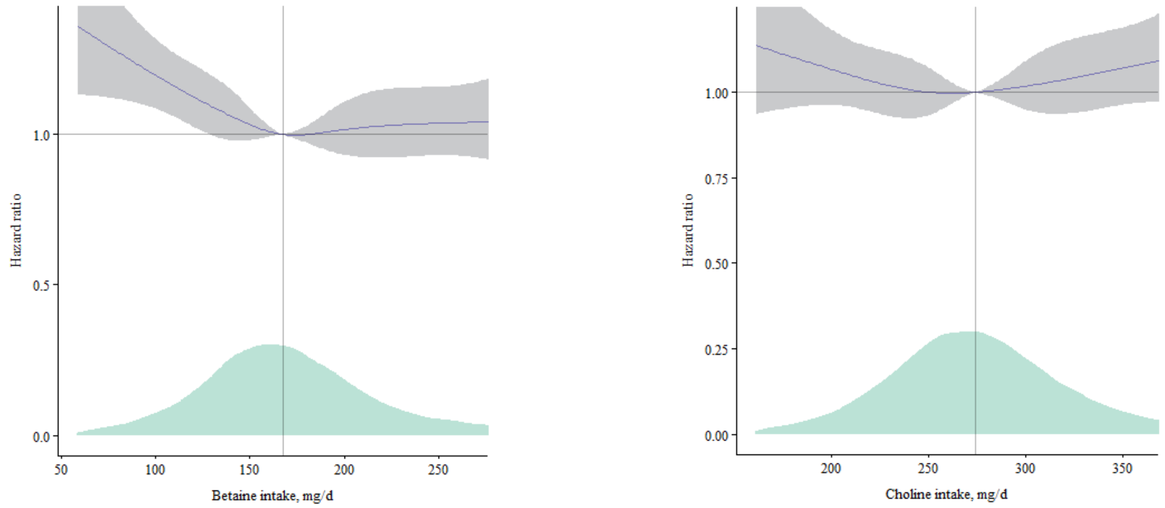

Supplemental Figure 2. Relation of betaine and total choline intake with risk of all-cause mortality in men. The solid lines show the observed association (hazard ratios) and the shaded areas 95% confidence intervals. Density plots indicate the distribution of choline and betaine intakes. The median was set as the reference. Model adjusted for energy intake (continuous), BMI (continuous), smoking (categorical), educational level (categorical) and physical activity (categorical). Choline and betaine intake is energy-adjusted using the residual method. Participants with intake the <1st and >99th percentiles were excluded from the analysis to minimize effects of extreme values.
